# Effectiveness of cognitive behavioral therapy on kinesiophobia and oral health-related quality of life in patients with temporomandibular disorders, study protocol for a randomized controlled trial

**DOI:** 10.1097/MD.0000000000023295

**Published:** 2020-11-20

**Authors:** Qi Zhang, Juan Zhang, Wenjing Ran, Shuipeng Yu, Yingshu Jin

**Affiliations:** a School of Nursing, Tianjin University of Traditional Chinese Medicine; b The TMJ Clinic, Stomatological Hospital of Tianjin Medical University; c School of Stomatology, Tianjin Medical University; d Department of Nursing, Stomatological Hospital of Tianjin Medical University, Tianjin, China.

**Keywords:** cognitive behavioral therapy, kinesiophobia, oral health related quality of life, randomized controlled trial, temporomandibular disorders

## Abstract

**Background:**

Temporomandibular disorders (TMD) is a common physical and psychological disease in dental department. Pain and mandibular limitation are the main reasons for patients to seek oral treatment. However, the presence of kinesiophobia, patients often catastrophize pain, so as to avoid mandibular movement, which seriously affects their quality of life. Cognitive behavioral therapy (CBT) has significant improvements in reducing kinesiophobia and quality of life in musculoskeletal disease, but has not been proved in TMD patients. The study aims to apply CBT on kinesiophobia and oral health related quality of life (OHRQOL) in TMD patients.

**Methods:**

A total of 108 individuals between 18 and 65 years of age, who will be referred to the temporomandibular joint clinic of Stomatology Hospital of Tianjin Medical University in china will be randomized into 2 treatment arms. The control group will receive a conventional treatment, whereas the experiment group will receive CBT on the basis of the control group. The primary outcomes will be the kinesiophobia and OHRQOL, and will be measured by the Tampa scale for kinesiophobia for patients with Temporomandibular Disorders (TSK-TMD) and the Oral Health Impact Scale for patients with temporomandibular disorders (OHIP-TMDs), the secondary outcomes will be pain intensity measured by Numerical Rating Scale (NRS), pain catastrophizing measured by Pain Catastrophizing Scale (PCS), anxiety and depression measured by Hospital Anxiety and Depression Scale (HADS), and self-efficacy measured by General Self-Efficacy Scale (GSES).

**Discussion:**

This study protocol reported a randomized controlled trial which aimed at assessing the effectiveness of the CBT versus conventional treatment with TMD.

**Trial registration:**

Registered in the Chinese Clinical Trial Registration Center with the number ChiCTR2000038573. Registered 24 September 2020.

## 1 Introduction

Temporomandibular disorders (TMD), is a general term for a class of diseases that involve temporomandibular joint (TMJ) and the corresponding soft tissues, which are characterized by muscle pain, abnormal mandibular function accompanied by dysfunction and abnormal joint sounds.^[1]^ Approximately 14.9% to 17.9% of the Chinese population suffered from TMD, and approximately 50% experience chronic pain.^[2,3]^ The early symptoms of TMD are relatively mild and easy to ignore, whereas the later symptoms are more severe, including restricted opening of the mouth, obvious pain, inability to chew normally, headaches, insomnia, dizziness, tinnitus, shoulder and neck pain, neurasthenia, irritability and other mental symptoms, which seriously affect daily life and physiological functions.^[4]^ The etiology of TMD is complex, which is believed to be the result of a combination of multiple factors. Many studies show that mental and psychological factors are the main factors that cause TMD, whereas pain intensity and fear of mandibular joint movement are the major reasons for TMD patients to seek oral treatment.^[5–7]^

Kinesiophobia, describes an excessive, irrational fear of physical activity that stems from susceptibility to painful injuries.^[8]^ Evidence shows that TMD has a pain amplification mechanism, patients are more sensitive to pain and the pain perception disappears for a longer time compared with the general population, and they take the necessary action to control pain and discomfort when experience it.^[9]^ For TMD patients, controlling the movement of the mandibular joint prevents the discomfort, therefore they avoid movement of the mandibular joint. The avoiding behaviors will be strengthened when the discomfort recurs, which is called to avoid positive feedback.^[10]^ This high level of vigilance against pain limits normal mandibular joint activities, which has a serious impact on the physical and mental health of patients.^[11]^ In addition to pain, Knapik believes that kinesiophobia may also be the fear of fatigue and physical symptoms, the fear of physical and mental discomfort, or the incoordination between the actual needs of the individual and the athletic ability.^[12]^

Physical activity and rehabilitation exercise are of great significance to functional recovery for chronic musculoskeletal diseases, such as chronic low back pain, fibromyalgia, knee pain, and other diseases.^[13]^ Functional recovery is the long-term treatment goal, besides the treatment of the injury, after bones and joints injury and the loss of limb function.^[14]^ Early exercise can increase blood circulation of the injured limb, restore the articular surface mobility, reduce joint stiffness and muscle atrophy.^[15]^ However, evidence show that kinesiophobia is an important predictor of disability in patients with acute or chronic low back pain,^[13]^ fibromyalgia,^[16]^ osteoarthritis,^[15]^ neck pain and other chronic musculoskeletal,^[17]^ leading to the occurrence of disuse syndrome.^[18]^

TMD have many similarities to chronic musculoskeletal diseases. Pain is the main complaint, but the affected area is limited to the maxillofacial region. Joint noise is a milder symptom in the early stage. In severe cases, the joint locking or jamming will appear, and patients will have total inability to open their mouth eventually.^[19]^ However, the incidence of joint locking is only 15%, most patients suffer from short-term function limitation. Conservative treatments are commonly used for TMD clinically. Among these therapies, mandibular exercise significantly relieve the disease symptoms.^[20]^ However, patients may avoid mandibular movement due to fear of pain, fatigue of the mandibular joint, and lack of correct coping ability. Long-term avoidance of joint movement causes positive feedback loops, This may affect the patient's eating, pronunciation, aggravating anxiety and depression, which has a serious impact on the patient's quality of life.^[21]^

Oral health-related quality of life (OHRQOL) is a comprehensive evaluation index that reflects the impact of oral diseases and prevention on patients’ physical, psychological, and social functions.^[22,23]^ Clarifying the OHRQOL is conducive to the choice of treatment, the detection of patient prognosis, as well as tracking oral risk factors.^[24,25]^ At present, the most common psychological problems that have been proved to affect TMD patients are anxiety, depression, and stress, but kinesiophobia didn’t get much attention.^[26,27]^ However, in the early stage of the research, the research group investigated the level of kinesiophobia and oral health-related quality of life (OHRQOL) in chronic painful TMD patients who were treated at Stomatological Hospital of Tianjin Medical University. The results show that the level of kinesiophobia in TMD patients is higher than the Korean TMD patients reported by Korean scholars.^[28]^ Kinesiophobia adversely affects patients’ oral function, psychophysiological status, and social activities, which can explain 75.2% of the variance in OHRQOL.^[29]^ Therefore, how to reduce kinesiophobia and improve the OHRQOL are the essential issues that should be considered in managing TMD.

Cognitive-behavioral therapy (CBT) refers to a psychological treatment to correct bad cognition by changing patients’ thinking, belief, and behavior, and to eliminate bad emotions and negative behaviors.^[30]^ It is characterized by integrity, initiative, enthusiasm, and a short course of treatment, and is suitable for patients without mental disorders.^[30,31]^ Studies show that CBT not only identifies and corrects errors in patients with automatic thinking and bad cognitive behavior, and reduces the patient's kinesiophobia, but also helps to enhance the patient's self-efficacy, alleviates anxiety and depression, improves physical activity and quality of life.^[32–35]^

However, to our knowledge, no previous studies have examined CBT for kinesiophobia in TMD patients. The purpose of this study is to apply CBT to TMD patients. Kinesiophobia and OHRQOL will be taken as the main outcomes, whereas pain intensity, pain catastrophizing, anxiety and depression, and self-efficacy will be taken as the secondary outcomes to explore the effects of CBT.

## 2 Methods

### 2.1 Study design

This study is a single blind randomized controlled trial with two parallel groups. This protocol was reported based on Standard Protocol Items: recommendations for Interventional Trials (SPIRIT) guidelines.^[36]^

### 2.2 Study setting

**Figure 1:**
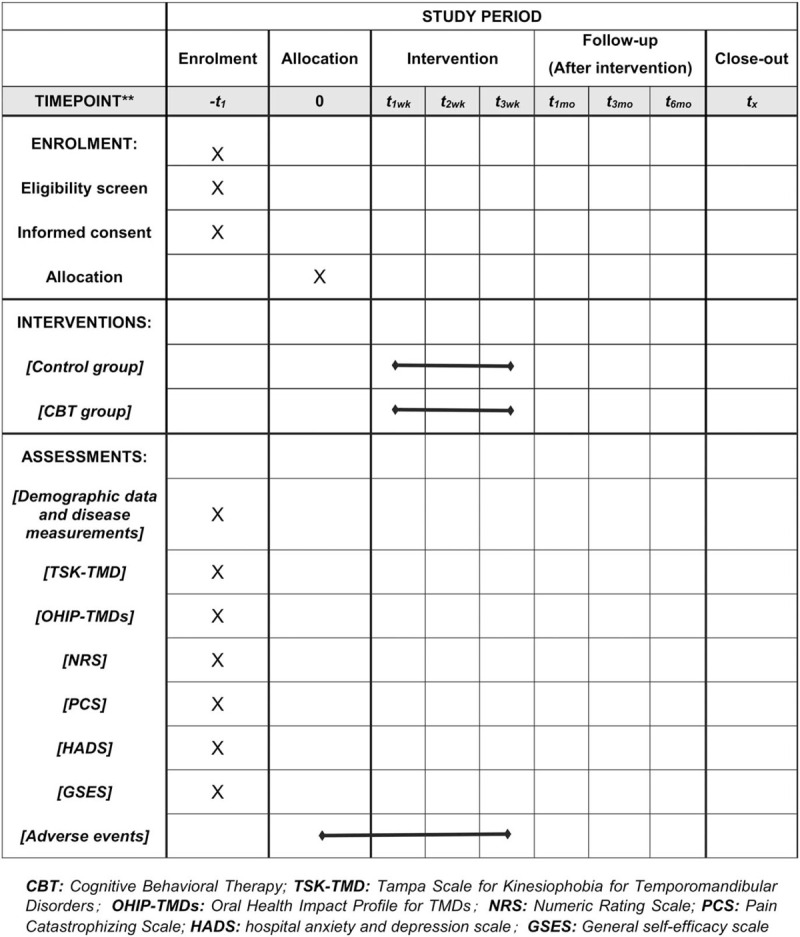
Standard protocol items: recommendations for Interventional Trials (SPIRIT) figure.

The study will be conducted at the TMJ clinic of the Stomatological Hospital of Tianjin Medical University in Tianjin, China. The participants will be informed the procedures, risks, benefits and the consent form, which will be conducted in accordance with the schedule described in Figure 1.

### 2.3 Participants

The Research Diagnostic Criteria Axis I for Temporomandibular Disorders (RDC/TMD-Axis I) will be adopted in the present study.^[37]^

### 2.4 Inclusion criteria

To participate in the study individuals must conform the following inclusion criteria:

1. Patients who meet the RDC/TMD-Axis I and are diagnosed with TMD;
2. Age between 18 and 65 years, can fill in the questionnaire independently and truthfully;
3. The chief complaint is accompanied by ≥3 months pain;
4. Voluntary participation research and fill in the informed consent form.

### 2.5 Exclusion criteria

Conversely, the exclusion criteria are:

1. Patients who have recently suffered from acute and chronic pulpitis, acute and chronic apical periodontitis, or suffered head and facial trauma;
2. Patients with a history of rheumatism and rheumatoid diseases;
3. Patients with a history of mental illness and drug addiction;
4. Patients with a history of heart and liver dysfunction or malignant tumors.

### 2.6 Interventions

#### 2.6.1 Control group

Conventional treatment will be delivered by one of the study dentist/TMD specialist. Usual treatment is conservative and typically include use of occlusal splints, non-steroidal anti-inflammatory medications, and patient education that comprise of passive and active range of mandibular movement exercises, modification of parafunctional and/or dietary habits and regular application of cold and heat packs.^[34]^ No attempt will be made to influence patient dental treatment.

#### 2.6.2 Experiment group

The experimental group will perform CBT on the basis of the control group. The CBT in this study will mainly be implemented by therapeutic meetings. The intervention times are the first visit and the second visit (the first and second weeks after the first visit), a total of three times, each meeting last about 30 minutes. The specific intervention steps and methods are as follows:

1. Establish an intervention team (before intervention)
  The study will establish an intervention group to ensure the frequent implementation. The members of this team include 1 TMD specialist, 1 nursing director, 1 doctor student (major in oral rehabilitation), 2 master students (major in dental nursing). The team will brainstorm and discuss, and then develop a pamphlet for interventions, which mainly includes the concept and mechanism of kinesiophobia, the relationship between fear-avoidance model, mandibular exercise, the importance of mandibular joint exercise. The site where the intervention will be implemented is the waiting hall of the TMJ clinic. Two members of the team will conduct cognitive and behavioral interventions on the participants. The members of the team should summarize the progress of the intervention of the research object in time, and communicate with each other to make the intervention plan proceed frequently.
2. Implementation of intervention measures (therapeutic meeting)
  - Session 1 (the first intervention)
    The main task of this session is to establish a good communication relationship with the patient and cognitive assessment. The researcher will choose the appropriate language and communication skills to communicate with patients so that they could reduce the sense of distance and establish a good cooperative relationship with participants, and then we understand the level of kinesiophobia in patients as well as the recognition of mandibular rehabilitation exercises, and record the related factors of kinesiophobia. Once we identify the patient's negative thoughts, we should make corresponding health education. At the same time, patients will be asked to scan the QR code of WeChat APP to obtain knowledge about TMD. After this session, it is necessary to summarize and provide feedback to determine whether the researcher and the patient's understanding are consistent.
  - Session 2 (the second intervention)
    First, the researchers will review the main content of the last meeting with the patient to understand their recent problems, so that they can prioritize the problems, and discuss, summarize and feedback on the important issues. Through the summary of the cognitive evaluation stage, the irrationality in thinking may be discovered by the researchers. For example, patients may have misconceptions such as “When I open my mouth, the pain will increase and the mouth will be painful”, “Joint exercises will cause joint deformities”, etc. Researchers should correct the patient's misconceptions in time, emphasizing that avoidance behavior can lead to joint capsule adhesion and long-term joint noise. In severe cases, it will be difficult to open the mouth and the pain will get worse. Early joint exercises are of great significance to restore mandibular function. Researchers should guide patients to divert attention and make positive changes, so that patients know that kinesiophobia is a problem to be solved rather than a decisive factor in life. By increasing the patient's confidence in overcoming the fear of exercise. At this stage, behavioral intervention can be used to reduce the patient's fear of exercise and relieve the patient's anxiety, depression and other unhealthy emotions.
  - Session 3 (the third intervention)
    The task of the third stage is cognitive reconstruction. The researcher will draft a complete long-term plan for the patient by checking the completion of the patient's target task, as well as affirm the patient's progress to further enhance the understanding of kinesiophobia and improve the patient's compliance with mandibular joint exercises. Finally, they should encourage patients to continue to perform relaxation therapy based on education manuals or videos, and explain the patients’ misunderstandings. After the end of the intervention, the participants will be thanked for their participation and cooperation in the study and encouraged to make regular follow-up visits. If they have any questions, they can contact us by WeChat APP to establish follow-up relationship.
  - Follow-up (1, 3, 6 months after the intervention)
    Researchers regularly push the knowledge about TMD and the protection of TMJ to patients through WeChat APP. During the first month of follow-up, it will push once a week, during the second to third months, once every two weeks, and once every month in the fourth to sixth month. The recovery of mandibular function will be asked regularly by researcher. The interventions will be described in Table 1.

**Table 1:** Checklist of CBT on TMD patients.

Intervention process	Intervention time	Intervention goal	Intervention content
The first stage	First visit	To establish a good relationship with the patient; cognitive assessment	①Understand the degree of kinesiophobia of the patient; ②Understand the relevant risk factors that cause kinesiophobia in patients; ③Provide WeChat QR code to facilitate patients to obtain TMD related knowledge; ④Establish short-term and long-term exercise goals based on the patient's terrorism and cognitive and cultural level.
The second stage	One week after the first visit)	Review the last conversation and get the latest news of the patient; correct unreasonable cognition	①Discover unreasonable disease thinking and cognition, and promptly correct the patient's “wrong cognition of thinking about kinesiophobia”; ②Explain the benefits of early joint exercises to patients; ③Encourage patients to overcome their kinesiophobia and continue to accomplish their goals.
The third stage	Follow-up consultation	Cognitive reconstruction	①Check the completion of the patient's rehabilitation goals and improve the exercise plan; ②Check the patient's mastery of relaxation training, find out the existing problems and give corrections and guidance; ③Encourage patients to perform rehabilitation exercises according to health education manuals and videos.
Follow-up	1, 3, and 6 month after the end of the intervention		①Regularly push TMD-related knowledge and how to protect the TMJ to patients through WeChat groups; ②The patients were followed up once a week in the first month after the intervention, every two weeks in the second to third months, and once a month in the fourth to sixth months, and patients were regularly asked about the improvement of pain and the recovery of mandibular function.

### 2.7 Outcome measures

The primary outcomes and secondary outcomes will be collected in 4 time points: before intervention, 1 month after intervention, 3 month after intervention, 6 month after intervention. All variables will be measured in both experiment group and control group.

### 2.8 Primary outcome measures

Kinesiophobia: The tampa scale for kinesiophobia for temporomandibular disorders (TSK-TMD) was developed by Visscher in 2010,^[21]^ and introduced to china by He et al to measure the status of kinesiophobia in TMD patients in 2016.^[38]^ The scale has 12 items and 2 dimensions, including activity avoidance and somatic focus. The scale uses the Likert 4-level scoring method, ranging from 1 (strongly disagree) to 4 (strongly agree), and the score ranges from 12 to 48 points. The high score indicates the high level of kinesiophobia. The Cronbach's α coefficient of the Chinese version of the scale is 0.919, and the Cronbach's α coefficients of the two dimensions are 0.895 and 0.907.^[38]^

OHRQOL: The oral health impact scale for patients with temporomandibular disorders (OHIP-TMDs) is a specific tool used to assess the OHRQOL of TMD patients.^[39]^ The Chinese version of the OHIP-TMDs was introduced by He et al in 2016.^[40]^ The scale consists 22 items, including 7 dimensions. Each item has 5 options, ranging from 0 (almost none) to 4 (very frequent), and the OHRQOL of TMD patients is evaluated from the aspects of limited function, physical pain, psychological discomfort, physical disorder, psychological disorder, social disorder, disability. The Cronbach's α coefficient of the Chinese version of OHIP-TMDs is 0.917, and the test-retest reliability is 0.899.^[40]^

### 2.9 Secondary outcome measures

Pain intensity: The numerical rating scale (NRS) is used for measurement.^[41]^ The scale divides a straight line into 10 parts, and uses the numbers 0–10 at each point to indicate the degree of progressive increase in pain, 0 means no pain and 10 means severe pain, Patients score themselves.^[42]^

Self-efficacy: The general self-efficacy scale (GSES) is a commonly used measurement tool to assess general self-efficacy.^[43]^ The GSES is single-dimensional, including a total of 10 items. It uses the Likert 4-level scoring method, ranging from 1 (completely incorrect) to 4 (completely correct), corresponding to 1 to 4 points. Total scores for the scale range from 10 to 40 points, where higher scores indicate higher self-efficacy. The Cronbach α coefficient of the Chinese version of the GSES is 0.87.^[44]^

Pain catastrophizing: Pain Catastrophizing is evaluated by pain catastrophizing scale (PCS), which is one of the most commonly used measurements with good reliability.^[45,46]^ It consists of 13 items with three dimensions: contemplation, magnification, and helplessness. Each item has 5 options, from 0 (not at all) to 4 (all the time). Total scores for the scale range from 13 to 52 points, where higher scores indicate higher pain catastrophizing. The effectiveness and reliability of PCS have been widely described in clinical and non-clinical samples.^[45]^

Anxiety and depression: Anxiety and depression are measured by the hospital anxiety and depression scale (HADS).^[47]^ The scale includes two subscales, for anxiety and depression, with 7 questions each. Each question includes 4 options, ranging from 0 to 3 points. The scores of the anxiety and depression subscales are divided into: 0–7 for asymptomatic, 8–10 for suspicious symptoms, and 11–21 for certain symptoms. When point ≥ 8 points, including suspicious and symptomatic, symptoms are all considered positive. The Cronbach α coefficient of the Chinese version is 0.762 and 0.787 respectively, indicating good reliability.^[48]^

### 2.10 Demographic information and clinical measurements

The study will use a self-designed general questionnaire, including general demographic information and clinical measurements. General demographic information includes the patient's sex, age, education level, and income level; The clinical measurements includes maximum mouth opening, joint noise, joint trauma, joint locking, mouth opening limitation, etc. The clinical measurements will be filled out by checking the patient's case book.

### 2.11 Sample size calculation

The sample size of this trial was based on the kinesiophoia score. Based on the previous research results of our research group, the mean of kinesiophobia was 34.37, with a standard deviation (SD) of 6.96 points.^[29]^ To achieve 90% power with a significant level of 0.05, a minimum of 90 patients is needed. Considering the possibility of withdrawal and loss of follow-up, the sample size was expanded by 20%, and the final sample size was determined to be 108 participants, with 54 participants in the experiment group and 54 participants in the control group. Sample Size was performed by using PASS Software (version 14.0; NCSS Statistical Software, Kaysville, UT) (http://www.ncss.com/software/pass/).

### 2.12 Patient recruitment

Recruitment for the study will begin in November 2020 and is expected to be completed in January 2020. Information about the purpose and process of the study will be provided orally by the researcher. The participants can ask questions or doubts about any aspect of the study. The investigator will provide participants with a guarantee of data confidentiality. The informed consent includes the background and purpose of the study, therapeutic interventions, research findings, and expected strengths and weaknesses.

### 2.13 Randomization and blinding

**Figure 2:**
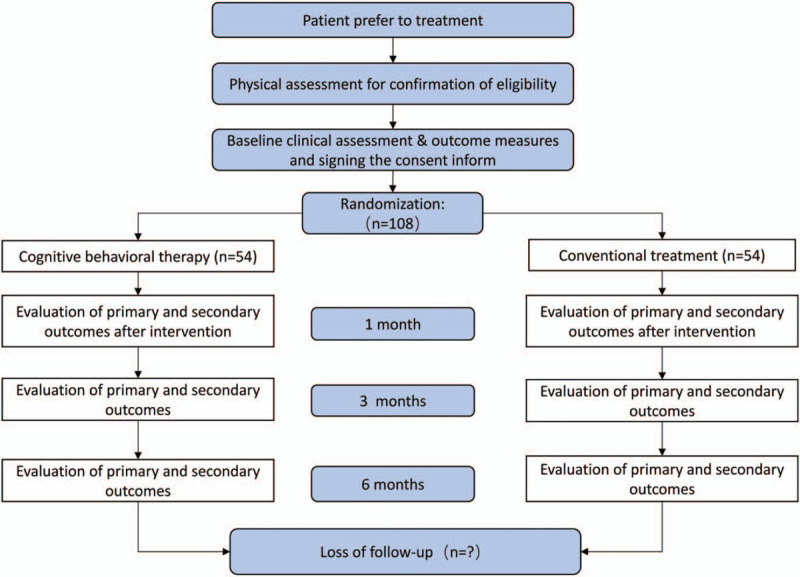
Study design.

The randomization of this study will be carried out by researchers who are not involved in recruitment. Participants will be grouped by the random number table method. Items assigned will be hidden and sealed in an opaque envelope in sequential numbered order. Participants will be randomly assigned to experiment group and control group. Before random allocation, they don’t know which treatment they would receive (Fig. 2).

### 2.14 Data management

The participants fill out questionnaires, and the information will be input into Excel for statistical analysis. None of the participants’ names are exposed during the study. All data in the trial will be stored in the computer and encrypted. Participants’ informed consent will be kept by the researcher.

### 2.15 Statistical analysis

SPSS21.0 software (IBM Corp., Armonk, NY) will be used to perform all statistical analysis. Continuous variables are described as the mean and standard deviation, and categorical variables are presented by percentages (%) and absolute (n) frequencies. In order to determine whether the parameters are suitable for statistical analysis, the normality test is firstly performed. Student's *t* test or chi-square test was to compare baseline data. Repeated-measures analysis of covariance (ANOVA) will be used to evaluate the effect of intervention on primary outcomes and secondary outcomes. *P < *.05 is considered statistically significant. Finally, the possible loss or withdrawal will be statistically analyzed according to the treatment intention.

### 2.16 Harms

Both groups of participants will make records during each treatment session in order to collect, evaluate, report and manage the potential adverse effects of the intervention in the study. According to informed consent, patients with increased symptoms after the treatment will be immediately evaluated by the TMJ specialists.

### 2.17 Ethics

The study will be conducted under the Declaration of Helsinki principles and standardized clinical practice. This study protocol has been approved by the Ethics Committee of Stomatological Hospital of Tianjin Medical University, with the reference number TMUhME2019001. The study has been registered in the Chinese Clinical Trial Registration Center with the number ChiCTR2000038573. If there is a need to modify this plan, the researchers need report to the Ethics Committee immediately. Participants need to acknowledge that they have participated in this study.

## 3 Discussion

Due to lack of research measurements and language limitation, the current studies mainly focus on epidemic, animal experiments in china. However, there is few studies conducted psychological behavior intervention on TMD patients, especially using kinesiophobia as the main outcome. To the best of our knowledge, this will be the first randomized controlled trial that we conduct a psychological intervention on Chinese TMD patients by using kinesiophobia and oral health related quality of life as primary outcomes to explore the effectiveness of CBT. The participants will be divided into experiment group and control group. The control group will receive a conventional treatment, whereas the experimental group will receive CBT on the basis of the control group. In previous studies, Cai et al^[35]^ conducted CBT on kinesiophobia to patients after total knee arthroplasty, kinesiophobia is significantly reduced after intervention, which ensured the amount of rehabilitation training for patients and promoted the recovery of knee function. Archer et al^[49]^ implemented CBT on patients after lumbar fusion surgery, and the results demonstrated that CBT can reduce kinesiophobia, as well as pain disability, the quality of life has also been significantly improved. However, the lack of individual or group treatment programs, the effectiveness of CBT for chronic pain is difficult to reach a definite conclusion.^[50]^

In previous studies, the efficacy of CBT in TMD patients has been explored, but most of these studies use pain intensity as a primary outcome, the duration of the effect has not been determined. The reason may be that there are great differences in CBT studies for TMD patients, including specific techniques, different background of the implementers, treatment frequency, treatment time, observation time after treatment, etc.^[51–55]^ In the study of the effectiveness of CBT alone, Mishra et al^[56]^ reported that patients in the CBT group had lower pain intensity compared to the non-treatment group, Gardea et al^[57]^ also reached the same conclusion. Dworkin et al^[52]^ reported that after 6 courses of CBT treatment, the patient's pain did not significantly improve compared with the conventional treatment group. Mishra et al^[56]^ performed more frequent CBT (12 courses of treatment) on patients without any mental disorders, and the pain intensity was significantly reduced. Tuner et al^[34]^ showed that CBT significantly improved the pain intensity in the 12-month follow-up, but haven’t been improved in the 8-week follow-up. These indicate that the intervention time and follow-up time need to be considered in the process of CBT.^[34,55]^

Through literature review, we found that the previous protocols of CBT mainly focused on the improvement of the negative emotions, such as anxiety, depression, and pain management, whereas our study focus on improving the patient's kinesiophobia, with the goal of improving patients’ quality of life. We correct the patient's poor cognition of the fear of mandibular movement, so that the patient can actively treat and exit the cycle of false cognition.

In addition, considering that the ability of CBT implementers may affect the patient's prognosis, the implementers of our research team will be trained by psychotherapists before the intervention. It will be carried out under the premise of comprehensively considering the patient's psychological status, disease severity, and treatment frequency.

To enhance the reliability of the results, methodological factors will be considered in our study. Taking the possible absence and withdrawal of the subjects into consideration, the sample size has been increased by 20%. If there is any missing or withdrawal of the participants, statistical analysis will be conducted according to the research protocol and intention-to-treat analysis.

Diagnostic and statistical manual of mental disorders (DSM)^[58]^ and international classification of diseases (ICD)^[59]^ has been recommended for psychiatric diagnosis. These manuals may contribute to the diagnosis of Axis II in TMD patients and the implementation of psychosocial interventions. However, the diagnostic criteria for Axis II are not widely used in China, and there are no clinical studies providing psychosocial treatment for TMD patients. However, the importance of axis II diagnosis and psychosomatic intervention should be recognized in managing TMD pain. Therefore, the clinical collaboration between TMD specialists and psychologists is essential to manage TMD.

It should be mentioned that the study has some limitations. First, this study will be only conducted in a specialized stomatological hospital with limited sample size, large sample size and multi-center randomized controlled trials can be carried out to verify the reliability of our study. Second, the outcomes of the study are mainly measured by self-report questionnaires. However, the differences generated by objective indicators are relatively small. Third, the study will only conduct CBT program, further research could combine exercise intervention, mindfulness intervention and other multi-component intervention treatment to explore the effects on kinesiophobia and OHRQOL in patients with TMD.

The study is a randomized controlled trial that aims to explore the CBT on kinesiophobia and OHRQOL in patients with TMD. The research results would provide supplements for the treatment and management of kinesiophobia, as well as provide evidence for controversial topics in TMD patients with CBT treatment.

## Author contributions

**Conceptualization:** Qi Zhang, Juan Zhang, Wenjing Ran, Yingshu Jin.

**Data curation:** Qi Zhang, Wenjing Ran, Shuipeng Yu, Yingshu Jin.

**Formal analysis:** Qi Zhang, Juan Zhang, Yingshu Jin.

**Investigation:** Qi Zhang, Wenjing Ran.

**Methodology:** Qi Zhang, Juan Zhang, Yingshu Jin.

**Project administration:** Qi Zhang, Juan Zhang, Yingshu Jin.

**Supervision:** Qi Zhang.

**Validation:** Qi Zhang, Wenjing Ran, Shuipeng Yu.

**Writing – original draft:** Qi Zhang, Wenjing Ran, Shuipeng Yu.

**Writing – review & editing:** Juan Zhang, Yingshu Jin.
